# Ruptured pheochromocytoma during preoperative doxazosin treatment

**DOI:** 10.1210/jcemcr/luag350

**Published:** 2026-11-11

**Authors:** Reema AlMaimoni, Abdullah AlFaraj, Osama AlSaif, Abdulaziz AlWosaibei, AlBatool Busbaih, Salma Hussein

**Affiliations:** Department of Internal Medicine, King Fahd Specialist Hospital, Dammam 32253, Saudi Arabia; Department of Endocrinology, King Fahd Specialist Hospital, Dammam 32253, Saudi Arabia; Department of Surgical Oncology, King Fahd Specialist Hospital, Dammam 32253, Saudi Arabia; Department of Endocrinology, King Fahd Specialist Hospital, Dammam 32253, Saudi Arabia; Department of Endocrinology, King Fahd Specialist Hospital, Dammam 32253, Saudi Arabia; Department of Endocrinology, King Fahd Specialist Hospital, Dammam 32253, Saudi Arabia

**Keywords:** ruptured pheochromocytoma, doxazosin, pheochromocytoma crisis

## Abstract

Rupture of pheochromocytoma is rare but represents a life-threatening complication. A 43-year-old man with episodic hypertension, palpitations, and chest discomfort was incidentally found, during a computed tomography (CT) aortogram performed to rule out aortic dissection, to have a 5.2-cm right adrenal mass suspicious for pheochromocytoma. Biochemical testing confirmed catecholamine excess. He was started on oral doxazosin 1 mg daily, which was titrated to 2 mg daily after 1 week, followed by oral bisoprolol 2.5 mg daily in preparation for surgery. Five weeks later, he presented with severe right-sided abdominal pain and a blood pressure of 215/111 mmHg. Abdominal CT angiography showed interval enlargement of the adrenal mass with posterior rupture and a retroperitoneal hematoma without active bleeding. He was stabilized with conservative management and subsequently underwent robotic right adrenalectomy. Histopathology confirmed pheochromocytoma with necrosis, without angioinvasion or lymphatic invasion, and with negative margins. Pheochromocytoma rupture can occur despite appropriate α-blockade, and spontaneous hemorrhage remains the most likely proposed mechanism, although α-blockade may be a contributing factor in selected cases.

## Introduction

Pheochromocytoma is a rare catecholamine-secreting tumor arising from adrenal chromaffin cells and is an important cause of secondary hypertension [1, 2]. Typical manifestations include episodic headache, palpitations, diaphoresis, and hypertension [3]. Rupture of pheochromocytoma is an uncommon but serious complication that may present with abdominal pain, hemorrhage, shock, or hypertensive crisis [4, 5]. We report a case of ruptured pheochromocytoma occurring after preoperative doxazosin therapy and discuss the possibility of a temporal association with α-blockade.

## Case presentation

A 43-year-old Saudi man, an ex-smoker, had a 5-year history of intermittent anxiety, palpitations, episodic anger, and recurrent elevated clinic blood pressure despite normal home readings. These episodes had initially been attributed to emotional stress. Over the preceding year, he developed chest tightness and was found to have blood pressure of 200/120 mmHg, prompting evaluation for aortic dissection.

A computed tomography (CT) aortogram excluded dissection but incidentally revealed a right adrenal mass. Dedicated adrenal CT showed a 5.2-cm heterogeneous right adrenal lesion suspicious for pheochromocytoma. He was referred to our tertiary center for further evaluation.

At presentation, he was normotensive and clinically stable. Plasma metanephrine and normetanephrine were markedly elevated, confirming catecholamine excess. Testing for hyperaldosteronism and Cushing syndrome was negative (Table 1). Repeat imaging approximately 7 months later showed further enlargement to 5.9 cm (Fig. 1), which, together with the biochemical findings, was consistent with pheochromocytoma. I-123 metaiodobenzylguanidine scanning showed no evidence of metastatic disease, and genetic testing for pheochromocytoma-paraganglioma-associated mutations, including *RET*, *VHL*, *NF1*, *SDHA*, *SDHB*, *SDHC*, *SDHD*, *SDHAF2*, *TMEM127*, and *MAX*, was negative. Preoperative preparation was initiated with doxazosin 1 mg daily, later titrated to 2 mg daily—the maximum tolerated dose given his baseline normotension—followed by β-blockade after 1 week. Blood pressure and heart rate remained well controlled during follow-up.

**Figure 1:**
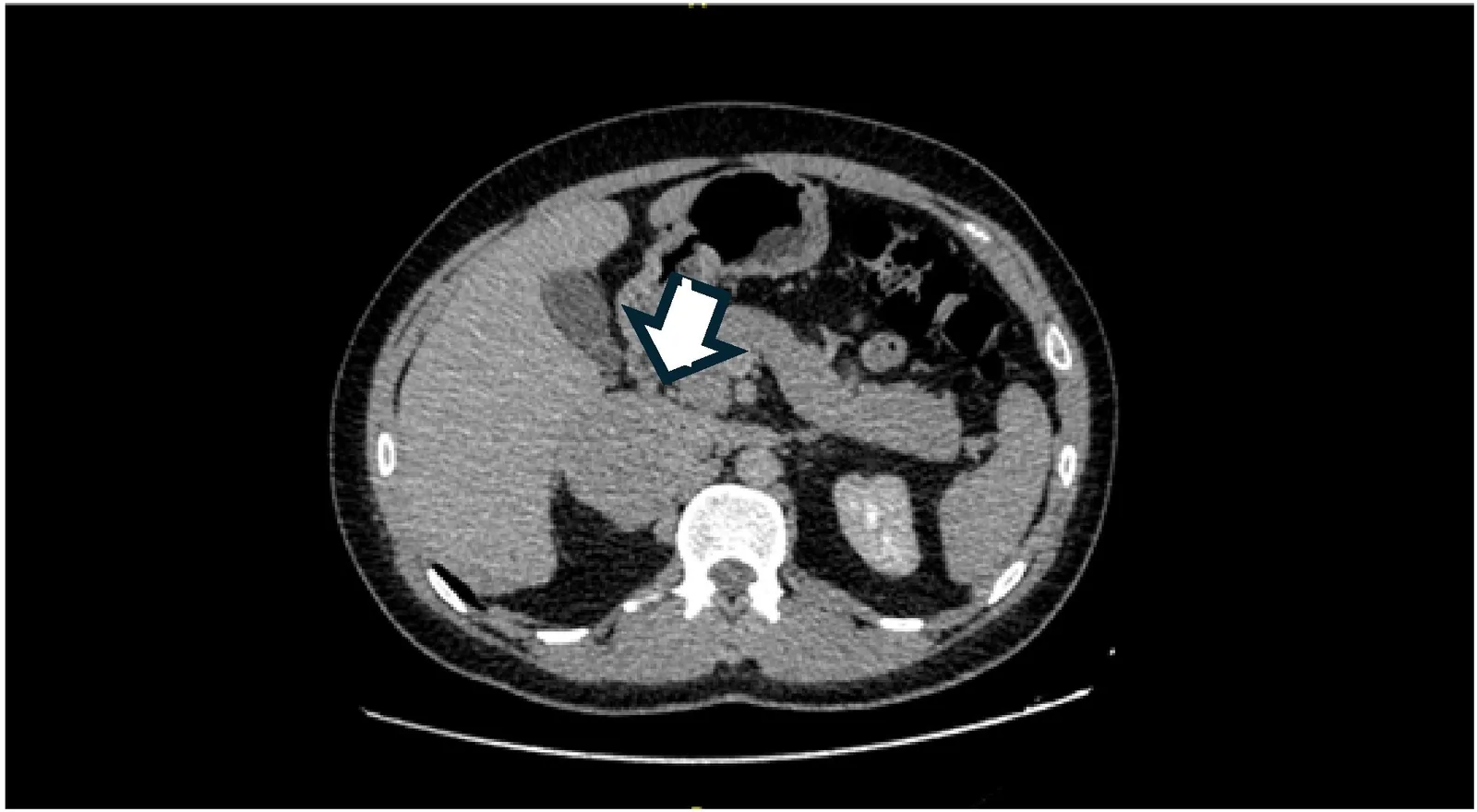
Contrast-enhanced computed tomography shows a 5.9-cm heterogeneous right adrenal mass (arrow) with cystic areas and no fat or calcification, without local invasion. Attenuation was 44 Hounsfield units (HU) precontrast, 94 HU portal venous, and 62 HU at 15 minutes, demonstrating absolute washout of 64% and relative washout of 34%, consistent with pheochromocytoma.

**Table 1:** Hormonal workup

Parameter	Result	Normal range
Plasma metanephrine (LC-MS/MS)	**420 pg/mL** **(SI: 2.13 nmol/L)**	<99 pg/mL (<0.50 nmol/L
Plasma normetanephrine (LC-MS/MS)	**>4500 pg/mL** **(SI: 24.56 nmol/L)**	<165 pg/mL (<0.90 nmol/L)
Aldosterone	**6.1 ng/dL** **(SI: 169 pmol/L)**	2.2 to 35 ng/dL (60-980 pmol/L) upright
Direct plasma renin concentration	525.5 mIU/L	3.4 to 56 mIU/L upright
Serum cortisol (post 1 mg dexamethasone suppression test)	0.70 µg/dL (SI: 19.3 nmol/L)	<1.81 µg/dL (<50 nmol/L)

Abnormal values are shown in bold font. Values in brackets are International System of Units (SI). Direct plasma renin concentration is reported per our lab unit.

Abbreviation: LC-MS/MS, liquid chromatography–tandem mass spectrometry.

## Diagnostic assessment

Five weeks later, he presented to the emergency department with sudden severe right-sided abdominal pain lasting 5 hours, associated with nausea and vomiting. He reported good adherence to doxazosin and bisoprolol. On examination, he appeared acutely ill. Blood pressure was 215/111 mmHg, heart rate 68 beats/minute, oxygen saturation 98% on room air, and temperature 36.8 °C. Abdominal examination showed marked right hypochondrial tenderness without guarding or rigidity.

Venous blood gas analysis showed metabolic acidosis with elevated lactate. Laboratory testing revealed mild leukocytosis and mild acute kidney injury. Liver function and electrolytes were otherwise unremarkable. Urgent abdominal CT angiography demonstrated interval enlargement of the right adrenal mass to 6.2 × 5.8 × 6.0 cm with posterior wall rupture and surrounding retroperitoneal, perirenal, and minimal subhepatic hematoma without active contrast extravasation (Fig. 2).

**Figure 2:**
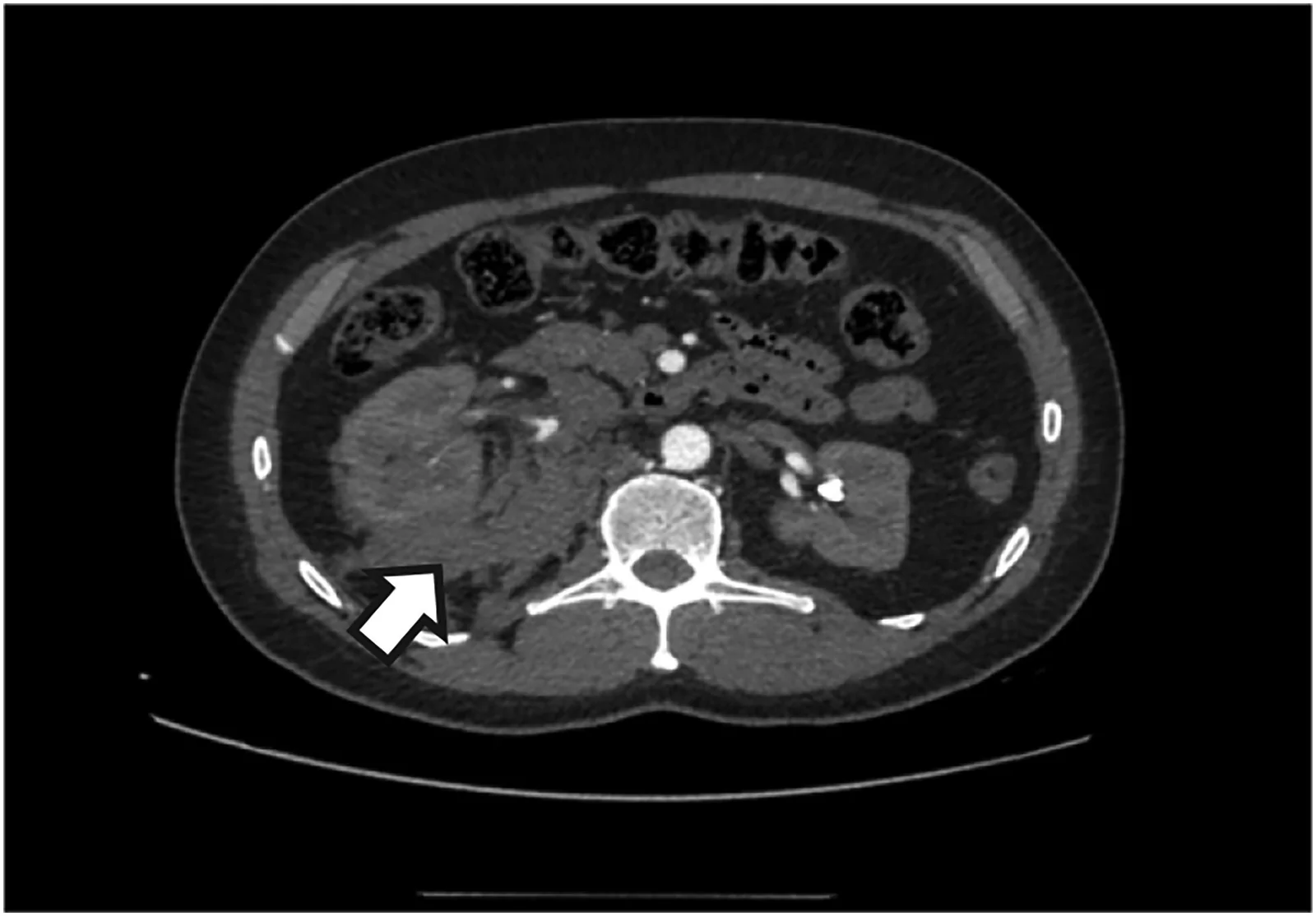
Contrast-enhanced axial computed tomography demonstrates a heterogeneous enhancing right adrenal mass consistent with pheochromocytoma, measuring 6.2 × 5.8 × 6.0 cm, with internal hyperdense components. A posterior wall defect (arrow) with surrounding hyperdense fluid (∼56 HU) extending into the right retroperitoneal, perirenal, and subhepatic spaces is noted, consistent with acute rupture and retroperitoneal hematoma.

## Treatment

The rupture occurred while he was awaiting elective robotic adrenalectomy. He was initially managed conservatively. Intravenous nitroglycerin was started at 10 µg per minute and titrated up to 50 µg per minute, resulting in effective blood pressure control. Nitroglycerin was chosen over phentolamine, nitroprusside, and nicardipine as these agents were unavailable in our facility. Because there was no evidence of active bleeding and the hematoma appeared contained, endovascular embolization was not pursued. Once he became hemodynamically stable, he was resumed on oral doxazosin 2 mg daily and oral bisoprolol 2.5 mg daily and was advised to increase dietary salt intake to support volume expansion prior to surgery. During the 2-day preoperative observation period, the hemoglobin was 16 g/dL (International System of Units [SI]: 160 g/L) (reference range, 13.8-17.2 g/dL [SI: 138-172 g/L]) and dropped to 11 g/dL (SI: 110 g/L), but he remained stable without evidence of ongoing hemorrhage. Two days after presentation, he underwent elective robotic right adrenalectomy (Fig. 3). The operation was completed successfully without major complications.

**Figure 3:**
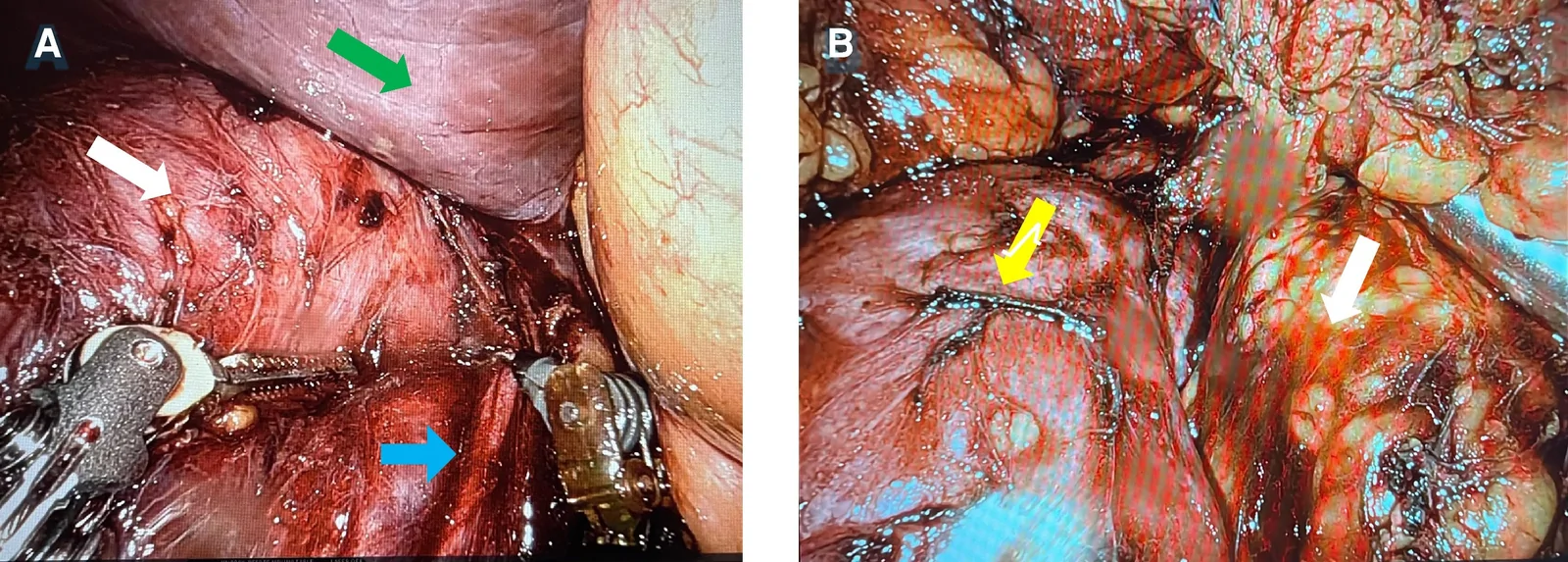
Intraoperative robotic view showing anatomical landmarks. (A) Pheochromocytoma (white arrow) and liver (green arrow) and inferior vena cava (blue arrow). (B) Pheochromocytoma (white arrow) and the kidney (yellow arrow).

## Outcome and follow-up

The final histopathology revealed a unifocal, intra-adrenal pheochromocytoma measuring 5.9 cm, with areas of tumor necrosis but no evidence of vascular or lymphatic invasion; the resection margins were negative, the Ki-67 proliferation index was 1%, and the tumor corresponded to pathologic stage pT2. The patient tolerated the adrenalectomy well and recovered without major complications. His abdominal pain and episodes of hypertensive crisis resolved completely after surgery, and he was discharged home normotensive without the need for antihypertensive medication. At the 4-month follow-up visit, the patient was asymptomatic, with blood pressure of 114/75 mmHg and a pulse rate of 88 beats/minute. Repeat plasma metanephrine and normetanephrine were 20.43 pg/mL (SI: 0.104 nmol/L) and 125.97 pg/mL (SI: 0.705 nmol/L), respectively. In view of the long-term risk of recurrent or metastatic disease, the patient was advised to undergo annual biochemical surveillance with plasma metanephrines.

## Discussion

Pheochromocytoma crisis is a life-threatening endocrine emergency resulting from the effects of catecholamine release on vital organ function. This entity occurs in approximately 3% to 18% of patients diagnosed with pheochromocytoma [4]. It can be precipitated by emotional stress, specific drugs, postural changes, surgery, and β-blockade administration without prior α-blockade [5]. Excessive catecholamine release causes severe hypertension, labile blood pressure, and extreme hyperthermia, resulting in multisystem end-organ injury. Reported complications include myocardial infarction, cardiomyopathy, acute heart failure, aortic dissection, acute respiratory distress syndrome, bowel ischemia or perforation, stroke, acute kidney injury, and lactic acidosis [5].

Ruptured pheochromocytoma is extremely rare but carries high morbidity and mortality due to the effects of catecholamine release [6]. Approximately 50 to 85 cases have been reported worldwide, of which, to the best of our knowledge, only one case has been reported in Saudi Arabia [7-9]. It was first described in the literature in 1944 by Cahill [10]. The precise underlying pathophysiology of pheochromocytoma rupture is unclear; however, it is thought to be related to increased intracapsular pressure from rapid tumor growth or intratumoral hemorrhage, leading to capsular tear. Consistent with this, our patient's tumor showed interval growth from 5.2 to 5.9 cm prior to rupture, supporting rapid tumor growth as a possible factor. The cause of ruptured pheochromocytoma is usually spontaneous; however, trauma and drug-induced causes, including thrombolytics, are described in the literature [11, 12]. The use of α-blockade, specifically phentolamine and phenoxybenzamine, has been reported in multiple case reports as a cause of ruptured pheochromocytoma through intratumoral hemorrhage and avascular necrosis. The proposed mechanism involves a decrease in systemic blood pressure, which compromises tumor perfusion and leads to necrosis, as the hemodynamic stability is subsequently restored, causing vasodilation and progressive intratumoral hemorrhage with eventual capsular rupture and hemoperitoneum. No such implication has been reported with doxazosin [13, 14]. Based on the existing evidence, hemodynamic instability, ruptured pheochromocytoma medically managed without surgical resection, cases reported prior to 1985, and incorrect perioperative diagnosis have been identified as independent factors of poor prognosis in ruptured pheochromocytoma [7].

The mainstay management for ruptured pheochromocytoma is surgical resection, which may be performed as an elective or emergent operation. Nonetheless, it is significant to start medical management with α-blockade for blood pressure control and fluid replacement, followed by elective or emergent surgical resection or by transcatheter arterial embolization followed by elective surgery.

Our case experienced pheochromocytoma rupture despite being on α-blockade with adequate blood pressure control. This raises the question of whether α-blockade reliably prevents pheochromocytoma-related crisis and rupture. Available evidence reported that routine preoperative α-blockade does not improve intraoperative hemodynamic stability in normotensive pheochromocytoma and may be associated with greater perioperative requirements for vasoactive support and intravascular volume replacement [15]. Current guidelines nonetheless recommend universal perioperative α-blockade for all patients; however, this recommendation is based on observational studies, as no randomized controlled trials are available given the low incidence of the disease [16]. While spontaneous rupture remains a possible cause in our report, a drug-induced mechanism related to α-blockade should also be considered, given the described association in the literature [13, 14].

## Learning points

- Ruptured pheochromocytoma is a rare but life-threatening cause of acute abdominal pain and hypertensive crisis.
- Conservative stabilization may be appropriate in selected hemodynamically stable patients without active bleeding.
- α-Adrenergic blockade remains standard preoperative therapy, but a temporal association with rupture has been reported.
- Definitive management requires multidisciplinary planning and timely adrenalectomy once stabilized.

## Contributors

All authors made individual contributions to authorship. R.M., A.F., A.W., A.B., and S.H. were involved in the diagnosis and management of the patient. O.S. was responsible for the patient's surgery. All authors reviewed and approved the final draft.

## Data Availability

Original data generated and analyzed during this study are included in this published article.

## Abbreviation

CT: computed tomography
HU: Hounsfield unit
LC-MS/MS: liquid chromatography–tandem mass spectrometry
SI: International System of Units

## References

[1] Reisch N, Peczkowska M, Januszewicz A, Neumann HPH. Pheochromocytoma: presentation, diagnosis and treatment. J Hypertens. 2006;24(12):2331‐2339. 10.1097/01.hjh.0000251887.01885.54 17082709

[2] Lenders JWM, Eisenhofer G, Mannelli M, Pacak K. Phaeochromocytoma. Lancet. 2005;366(9486):665‐675. 10.1016/S0140-6736(05)67139-5 16112304

[3] Falhammar H, Kjellman M, Calissendorff J. Initial clinical presentation and spectrum of pheochromocytoma: a study of 94 cases from a single center. Endocr Connect. 2018;7(1):186‐192. 10.1530/EC-17-0321 PMC5776668 29217652

[4] Luo S, Cui Q, Wang D. Case report: surgical intervention under pheochromocytoma multisystem crisis: timing and approach. Front Oncol. 2022;12:908039. 10.3389/fonc.2022.908039 PMC9252594 35795034

[5] Mazza A, Armigliato M, Marzola MC, et al Anti-hypertensive treatment in pheochromocytoma and paraganglioma: current management and therapeutic features. Endocrine. 2014;45(3):469‐478. 10.1007/s12020-013-0007-y 23817839

[6] Hanna JS, Spencer PJ, Savopoulou C, Kwasnik E, Askari R. Spontaneous adrenal pheochromocytoma rupture complicated by intraperitoneal hemorrhage and shock. World J Emerg Surg. 2011;6(1):27. 10.1186/1749-7922-6-27 PMC3178469 21843357

[7] Kobayashi T, Iwai A, Takahashi R, Ide Y, Nishizawa K, Mitsumori K. Spontaneous rupture of adrenal pheochromocytoma: review and analysis of prognostic factors. J Surg Oncol. 2005;90(1):31‐35. 10.1002/jso.20234 15786413

[8] Bhandarwar AH, Tandur AE, Rachapalli KR, Wagh A, Shah A, Dhimole N. Minimally invasive management of hemorrhagic pheochromocytoma: a rare case report. Surg J (N Y). 2023;9(1):e52‐e57. 10.1055/s-0043-1762554 PMC10040260 36987408

[9] Ahmed EM, Alshammari AN, Alharbi BS, et al Spontaneously ruptured pheochromocytoma with retroperitoneal hematoma mimicking acute myocardial infarction and acute pancreatitis. Med Sci. 2020;24(103):1439‐1444.

[10] Cahill GF . Sympathoblast. Pheochromoblast. Penn Med J. 1944;47:664‐667.

[11] Souiki T, Tekni Z, Laachach H, et al Catastrophic hemorrhage of adrenal pheochromocytoma following thrombolysis for acute myocardial infarction: case report and literature review. World J Emerg Surg. 2014;9(1):50. 10.1186/1749-7922-9-50 PMC4177258 25276225

[12] May EE, Beal AL, Beilman GJ. Traumatic hemorrhage of occult pheochromocytoma: a case report and review of the literature. Am Surg. 2000;66(8):720‐724. 10966025

[13] Vasudeva Pai H . Rupture of pheochromocytoma caused by phenoxybenzamine: a case report. Urol Case Rep. 2018;20:70‐71. 10.1016/j.eucr.2018.07.005 PMC6043816 30009134

[14] Van Way CW 3rd, Faraci RP, Cleveland HC, Foster JF, Scott HW Jr. Hemorrhagic necrosis of pheochromocytoma associated with phentolamine administration. Ann Surg. 1976;184(1):26‐30. 10.1097/00000658-197607000-00004 PMC1344300 938114

[15] Shao Y, Chen R, Shen ZJ, et al Preoperative alpha blockade for normotensive pheochromocytoma: is it necessary? J Hypertens. 2011;29(12):2429‐2432. 10.1097/HJH.0b013e32834d24d9 22025238

[16] Fang F, Ding L, He Q, Liu M. Preoperative management of pheochromocytoma and paraganglioma. Front Endocrinol (Lausanne). 2020;11:586795. 10.3389/fendo.2020.586795 PMC7551102 33117294

